# Acute mastoiditis caused by Klebsiella pneumoniae carbapenemase (KPC) producing bacteria – an atypical cause 

**DOI:** 10.3205/dgkh000619

**Published:** 2026-02-06

**Authors:** Aristos Aristodimou, Zacharias Raptopoulos, Georgios Georgiou, Elena Xenofontos, Loukia Dramiotou

**Affiliations:** 1 Internal Medicine Department, Limassol General Hospital, State Health Organization Services, Limassol, Cyprus; 2 Ear – Nose – Throat (ENT) Department, Limassol General Hospital, State Health Organization Services, Limassol, Cyprus

**Keywords:** acute mastoiditis, carbapenemase producing Klebsiella pneumoniae, KPC, immunosuppression

## Abstract

**Aim:**

The aim of this report is to present a rare case of acute mastoiditis caused by *Klebsiella (K.) pneumoniae* carbapenemase (KPC) producing bacteria.

**Methods:**

The following report describes a 78-year-old immunosuppressed female who was hospitalized due to left-sided acute otitis media complicated by tympanic membrane rupture and acute mastoiditis. Fluid-discharge culture revealed KPC. The patient successfully received four weeks of conservative treatment with intravenous colistin (based on antibiotic susceptibilities).

**Discussion:**

Acute mastoiditis is a rare complication of acute otitis media, mostly seen in pediatric populations. The most frequent causative agents identified are Gram-positive cocci such as *Streptococcus (S.) pyogenes* and* S. pneumoniae*. Acute mastoiditis caused by KPC-producing bacteria has rarely been reported in medical literature, with most such infections observed following neurosurgical surgeries and/or post-trauma.

**Conclusion:**

The incidence of infection by KPC is rising, especially in immunosuppressed patients and those with recent and/or prolonged hospitalizations. There are very few reports in the literature describing acute mastoiditis caused by KPC. Infection with KPC not only has a high 30-day mortality rate (up to 40%) but also poses a significant financial burden on the healthcare system.

## Zusammenfassung

**Ziel:**

Ziel des Berichts ist die Darstellung des seltenen Falls einer akuten Mastoiditis, verursacht durch einen Carbapenemase bildenden *Klebsiella (K.) pneumoniae* (KPC).

**Methode:**

Es wird eine 78-jährige, immunsupprimierte Patientin beschrieben, die aufgrund einer linksseitigen akuten Otitis media mit der Komplikation einer Trommelfellperforation und akuter Mastoiditis hospitalisiert wurde. Die Untersuchung des Ohrsekrets ergab das Vorhandensein von KPC. Die Patientin wurde erfolgreich über einen Zeitraum von vier Wochen konservativ mit intravenösem Colistin (basierend auf dem Antibiogramm) behandelt.

**Diskussion:**

Die akute Mastoiditis ist eine seltene Komplikation der akuten Otitis media, die am häufigsten in der pädiatrischen Population vorkommt. Die häufigsten identifizierten Erreger sind Gram-positive Kokkenbakterien wie *Streptococcus (S.) pyogenes* und *S. pneumoniae*. Eine durch KPC verursachte akute Mastoiditis wurde in der medizinischen Literatur bislang nur selten beschrieben, meist im Zusammenhang mit neurochirurgischen Eingriffen oder posttraumatischen Zuständen.

**Schlussfolgerung:**

Die Inzidenz von Infektionen mit KPC nimmt zu, insbesondere bei immunsupprimierten Patienten sowie bei Personen mit kürzlichem und/oder längerem Krankenhausaufenthalt. In der Literatur finden sich nur sehr wenige Berichte über akute Mastoiditiden infolge einer KPC-Infektion. Infektionen mit diesem Erreger gehen nicht nur mit einer hohen 30-Tage-Mortalitätsrate (bis zu 40%) einher, sondern stellen auch eine erhebliche finanzielle Belastung für das Gesundheitssystem dar.

## Introduction

Acute mastoiditis is characterized by inflammation of the mastoid air cells and often arises as a complication of acute otitis media infection [1]. It is most of seen in pediatric populations [2], whereas the incidence in adults is only about 0.99/100,000 [2] and can present as either the acute, classical form, or the latent form followed by rapid deterioration [3]. Other atypical causes of acute mastoiditis include hematogenous spread, or entry of nasopharyngeal secretions into the middle ear via the Eustachian tube, due to aspiration, insufflation or reflux [4]. Symptoms include otalgia, otorrhea, headache and, in advanced cases, several intracranial complications [5]. Upon clinical examination, retro-auricular swelling and erythema with associated tenderness can be observed [5]. The diagnosis is based on clinical symptoms such as presence of fever, otalgia, protrusion of the auricle and post-auricular erythema, tenderness, swelling, fluctuance or mass, but imaging techniques can be used as adjuncts in case the clinicians suspect complications [2], [6]. Cultures of fluid drained from the affected ear are positive around 58% of the time and the most common pathogens associated with acute mastoiditis include *Streptococcus (S.) pneumoniae*, *S. pyogenes* and *Pseudomonas aeruginosa* [5]. *Klebsiella (K.)* spp. is a rare cause of acute otitis media (3.9% prevalence in children) and even more rarely as a cause of acute mastoiditis [7]. In this report, we describe the case of a patient who was diagnosed with left-sided, acute otitis media complicated by tympanic membrane rupture and acute mastoiditis. The causative agent identified was carbapenemase-producing *K. pneumoniae* (KPC), which has been only rarely reported in literature. 

## Case presentation

A 78-year-old female with a medical history of chronic lymphocytic leukemia (CLL), diabetes mellitus type 2 (DM II), polymyalgia rheumatica (PMR), chronic kidney disease (CKD) stage 4 (eGFR 29), hyperuricemia, hypertension, asthma, hyperlipidemia and hypothyroidism visited the emergency department with a one-week history of left-sided earache associated with otorrhea. She denied experiencing any fever, nasal congestion, facial pain, dizziness, vertigo, or any other systemic symptoms. The patient is a non-smoker and does not consume any alcohol or illicit drugs. She denied traveling abroad during the past year. The patient was recently admitted to the pulmonology ward (one month before presentation), due to SARS-CoV-2 pneumonia and *Pneumocystis jirovejii* infection. During that period, the patient received treatment with piperacilin/tazobactam, ciprofloxacin, meropenem, doxycycline, remdesivir and trimethoprim/sulfamethoxazole. At that time, bronchoscopy was done, and broncho-alveolar lavage (BAL) culture was negative for *Klebsiella* spp. The film array was positive only for rhinovirus. Blood cultures were also negative. The patient had not been previously screened for colonization by KPC. 

Upon presentation, she was afebrile (36.6°C) and hemodynamically stable (115/55 mmHg), normal cardiovascular and respiratory system status, orientated to time, place and self, without any focal neurological signs upon examination. There was erythema and tenderness in the posterior auricular area of the left ear but no bulging. At the emergency department, an ear-nose-throat (ENT) consultation was requested, and upon evaluation, a perforated left tympanic membrane was noted along with purulent discharge, in the absence of erythema of the external auditory meatus. Fluid was collected through aspiration from the perforation site and sent for analysis that later returned positive for KPC, sensitive only to colistin.

Blood tests were requested and revealed leukocytosis (31,980 cells/µL), normocytic anemia (hemoglobin 10.4 g/dL with mean corpuscular volume of 98.5 fl), elevated serum creatinine levels (creatinine 1.70 mg/dL) and elevated CRP (33.14 mg/L). Blood cultures were negative. Based on the clinical findings, there was high suspicion of acute mastoiditis. A CT brain/petrous temporal bone was requested, which revealed complete opacification of the mastoid air cells on the left with associated erosion of their bony septate, consistent with acute coalescent mastoiditis. The patient was started on antibiotic therapy with colistin and meropenem for four weeks. Since the tympanic membrane was perforated, no draining tube was placed. During hospitalization, the patient was reviewed by the ENT specialist on multiple different occasions with evidence of significant improvement on examination of the left ear: adequate drainage was noted. Repeated audiograms revealed no hearing loss. The patient completed four weeks of antibiotic therapy and was discharged after showing significant improvement. A follow-up evaluation in the ENT outpatient clinic a month later showed restoration of the ruptured tympanic membrane and a normal audiogram.

## Discussion

Acute mastoiditis is most commonly caused by *S. pneumoniae*, S. pyogenes, coagulase-negative staphylococci, *Haemophilus influenzae*, *Turicella otitidis* and anaerobic bacteria [7]. *Klebsiella* is an uncommon cause of acute otitis media or acute mastoiditis [8], [9]. It is found on solid surfaces and water, but it also colonizes the intestines and upper respiratory tract of humans [9]. *Klebsiella* spp. has been described as a typical opportunistic pathogen, often encountered in hospital settings, and it has been found accountable for about one-third of all Gram-negative infections. In the community, it can cause endogenous endophthalmitis, pyogenic liver abscesses, and necrotizing pneumonia. It belongs to the ESKAPE category of organisms that are defined by their ability to resist or evade the facets of antimicrobial drugs [9]. 

KPC poses an increasing worldwide danger, with data suggesting a pooled prevalence of 5.43% and a pooled incidence 22.3% [10]. Only a handful of case reports describe KPC as the causative agent of acute mastoiditis. Baharoon et al. [8] reported a case of left-sided mastoiditis and skull-based osteomyelitis caused by KPC. Anwar et al. [11] published a case report of KPC-induced meningitis, brain abscess, and left-sided otomastoiditis. A recent article published by Babich et al. presented the case of an immunosuppressed patient with right-sided acute mastoiditis, caused by New-Delhi metallo-ß-lactamase (NDM) *K. pneumoniae* [12]. 

Carbapenemases are serine β-lactamases that use carbapenems as hydrolysis substrates [10], [13]. Such proteins are encoded by multiple genes, with the most common ones being KPC2, KPC3, VIM–1, IMP-4, NDM–1, NDM–5, OXA–48, and OXA–181 [10]. Certain risk factors, such as diabetes mellitus, chronic obstructive pulmonary disease (COPD), immunocompromised state, malignancy, renal insufficiency, and residency at skilled nursing facilities predispose to colonization and infection with KPC, with a high mortality rate [11]. Papadimitriou-Olivgeris et al. [14] mentioned that previous treatment with certain antibiotics, such as carbapenems and β-lactams/β-lactamase inhibitors, constitute another significant risk factor for colonization with KPC. Relevant to the case presented, the patient had several risk factors putting her at risk for colonization with KPC, such as previous admission to a hospital and treatment with meropenem and piperacilin/tazobactam, as well as significant comorbidities, including CLL, CKD4, and DM. According to the most recent guidelines, first-line treatment choices include ceftazidime/avibactam (CAZ/AVI), with restoration of susceptibility to ceftazidime up to 80%, and meropenem/vaborbactam (MVB) with 60–75% clinical success rates [15], [16], [17]. In contrast, the patient presented above received colistin, guided by the susceptibilities listed on the antibiogram, in addition to meropenem.

To recapitulate, KPC is a very rare cause of acute mastoiditis [8], [11], [12]. The rising number of immunosuppressed patients – estimated to be around 6.6% in the US (2.3% in 2013) – along with the increasing prevalence of KPC (and other MDR) pose a significant danger for colonization by such bacteria [10], [18]. This could mean a higher number of infections caused by them, resulting in significant morbidity and mortality; the latter 30-day rate is about 40% [19]. Finally, treatment for MDR, and more specifically KPC, has significantly higher costs to the healthcare system during the period of infection [19]. Mobaque dos Santos et al. [19] reported a 72% higher cost during the period of infection with KPC compared to other periods.

## Conclusion

The incidence of infection by KPC is rising, especially in immunosuppressed patients and those with recent and/or prolonged hospitalizations. There are very few reports in the literature describing acute mastoiditis caused by KPC. Infection with KPC not only has a high 30-day mortality rate (up to 40%) but also poses a significant financial burden on the healthcare system.

## Notes

### Authors’ ORCID:

- Aristodimou A: https://orcid.org/0009-0003-6567-1999
- Raptopoulos Z: https://orcid.org/0009-0008-4798-9203

### Funding

None.

### Competing interests

The authors declare that they have no competing interests.
